# Jacques Joseph: Father of modern aesthetic surgery

**Published:** 2008-10

**Authors:** Surajit Bhattacharya

**Affiliations:** Sr. Consultant, Lucknow Plastic Surgery, Lucknow, India

**Keywords:** Jacques Joseph, history of modern aesthetic surgery

## Abstract

When we review the history of modern aesthetic surgery, a name that stands out as bright as a beacon and precious as gold is undoubtedly that of Jacques Joseph. A surgeon, par excellence, far ahead of his time, who chose to think out of the box, Joseph, despite all odds set out to give respectability to Aesthetic Surgery without depriving it of any scientific core values. By his words and deeds proved beyond doubt that only the very best in the field of reconstructive surgery, can visualize the hidden perfection in imperfection and formulate a treatment plan and a surgical strategy to achieve that elusive perfection. The rich surgical literature that he has left behind, the wealth of surgical instruments that he had designed and above all a way of thinking that he propagated, that aesthetic surgery is not frivolous but very serious endeavor, and treating the psychology of the patient is as important as treating his disease, undoubtedly makes him the revered ‘Father of Modern Aesthetic Surgery’.

## INTRODUCTION

Though the history of Plastic Surgery is as old as the era of Sushrutha around 700BC, modern Plastic Surgery began about a century ago when a few brave surgical warriors began exploring a new frontier where reconstruction of body parts lost in injury and in battle was tried with an aim to restore both form and function. These, the then new age surgeons, were trained in different specialties, including otolaryngology, general surgery and orthopaedics and so brought to their work different knowledge and skills from their parent specialities. They interacted with each other, observed one another and were soon able to both expand the repertoire of their work and the finesse in it. These were the newer generation of Plastic and Reconstructive Surgeons.

**Figure 1:**
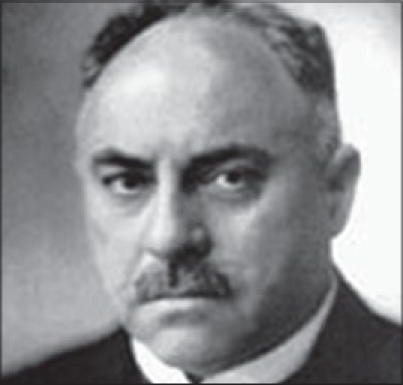
Jacques Joseph (September 6, 1865 -February 12, 1934) (Source: http://www.aafprs.org/patient/about_us/h_father.html)

Out of this group of dedicated new generation specialists one surgeon in particular stands out from this time. Jakob Lewin (Jacques) Joseph [Figure 1] was born on 6^th^ September 1865 in Königsberg. He was the third child of the Rabbi Israel Joseph and his wife Sara. From 1885 to 1889 he studied medicine at the Friedrich-Wilhelm University in Berlin. He completed his studies in 1889 and obtained his doctorate in 1890 in Leipzig.[1]

### How the magic began

After the stipulated period of his medical licensing and medical practical training in 1892, Joseph became a general practitioner in the district of Berlin-Mitte. Though he did exceptionally well in his practice a desire to specialize always kindled inside his heart. In 1892 he applied to the University Polyclinic to work in Orthopaedic Surgery and was selected for training. The Unit was headed by Professor Julius Wolff (1836-1902) – a recognized surgeon, called ‘Knochenwolff’ (Bonewolf) by the Berlin populace. Dr. Wolff, who is considered one of the pioneers of modern orthopaedics, liked young Joseph and took keen interest in his training. This relationship of mutual respect between the teacher and the taught however came to an abrupt end one day when Joseph, true to his nature, chose to think out of the box. Joseph undertook to surgically correct the ears of a ten-year-old boy, who refused to attend school because he suffered such ridicule from classmates for his large, protruding ears, which were too large and stuck out much too far (‘donkey's ears’). Though Joseph was not sure whether such surgery had ever been performed in the past, he felt it was possible and after careful planning he operated upon the child successfully. He took his success story to the Berlin Medical Society and was highly appreciated by his peers but this show of originality also cost him his job with Wolff, who felt that Joseph had risked the reputation of Wolff's clinic by performing the maverick procedure. Thus, after training with Wolff for 4 years, a training which if completed could have earned him a University degree, in 1896 Joseph was back to square one and he returned to private practice.[2]

### Conceptualizing Aesthetic Surgery

Two years later, a man with a very large nose approached Joseph and pleaded that he was too embarrassed to be seen in public because of his nose and he should do something about it. The man had heard about the ear reduction Joseph had performed and wondered, if he could reduce a lager pair of ears, then why could he not do the same for his nose?

Joseph too felt that this was possible, but as he had never thought about it he thought should study the anatomy of the nose further. He practiced his nose reduction surgery on cadavers and later on operated on the patient, much to the latter's satisfaction. Again he reported his success in May 1898 to the Berlin Medical Society, but this time with the case report he propounded a landmark theory.

Joseph had been developing this theory for quite some time, and it postulated that the psychological aspect of aesthetic surgery was as important as its physical success. According to the theory, a person whose looks caused social or economic disadvantage was as severely afflicted as a person who suffered from a debilitating disease. Now considering that the year was 1898, this theory was way too radical for all those purists of his era as it was outside the mainstream of the then serious curative medicine. The “serious” surgeons were in a habit of scorning the use of their skills for petty cosmetic purposes. It was also contrary to Joseph's Prussian background, which sternly admonished one to make do with what life dealt. Joseph called the desire to look normal “anti-dysplasia” not vanity.[3] Hence for his time and society his was a very bold step towards authenticating aesthetic surgery. So there is little doubt why the courageous Jacques Joseph today is considered the father of modern aesthetic surgery!

### The First World War

When the First World War started Joseph, by then a reputed facial plastic surgeon amongst both his peers and his patients, was entrusted with responsibility of staff physician in reserve. This was a whole new war with newer and more lethal ammunitions which would inflict injuries of a frequency and severity unknown until then. Jacques Joseph, rose up to the newer challenges and fired by his patriotic sense of service to the German fatherland increased the number and extent of his operations to the most extreme degree. As it was expected because of his grasp of the fundamentals of facial surgery, he worked with extraordinary success in the field of reconstructive surgery as well, attaining quite spectacular successes. It is said that the ‘Supreme Commander’ Wilhelm II himself came to notice him, or at least Joseph was brought to his attention for his extraordinary services.[3]

Joseph had no desire to chair any department but in 1915 the Emperor personally offered him the Chair for Plastic Surgery at the Charité hospital – but only under the condition that he, Joseph, convert to Christianity. Joseph refused. He was proud of his Jewish heritage but why the Supreme Commander made this extraordinary offer is not very clear. Did he want to quiet his conscience by offering the wounded soldiers, some of whom were horribly disfigured, the ‘best facial surgeon in the world’ in such an exposed position? Then again did such scruples and sentimentality come naturally to Wilhelm II perhaps no one would ever know!

On 2 June, 1916 the Prussian Ministry for Ecclesiastical and Educational Matters (Ministerium für geistliche und Unterrichtsangelegenheiten) gave Joseph a Department of Facial Plastic Surgery, which was opened at the Ear and Nose Clinic of the Charité, then headed by Adolf Passow (1859-1926). This was a post which carried no remunerations for Joseph. Three years later in 1919 he was named professor – but this time not by the Emperor, and without any impossible religion conversion conditions being attached. During his time at the Charité he accomplished great reconstructive feats using regional or forehead and upper arm flaps. He also performed plastic surgery on one hand, and free cartilage and bone transplants on the other. His bulk of work remained facial plastic surgery and he succeeded in reconstructing faces even in cases of extensive injury. His War time services earned him the Iron Cross.[3]

### Nasen Joseph in Private Practice

In 1922 the Army had no further need for Joseph's services as the war victims were mostly well managed or dead by then. They stopped financing his department and Joseph returned to his practice. This proved to be a boon in disguise and he dedicated himself increasingly to corrective and aesthetic surgery. In 1922 a report on Jacques Joseph was published by “raving reporter” Egon Erwin Kisch. A much reputed and respected surgeon by now, most of his work was corrective procedures for the nose and ‘hanging cheeks’, as well as breast operations. He was known as ‘Nasen-Joseph’ (Nose-Joseph) or ‘Noseph’ by the Berliners, such was the popularity of his rhinoplasties! He attracted the newer generation of Plastic surgeons like a magnet and his Unit almost always had visitors from both Europe and America. One of his famous assistants Gustave Aufricht, later went to New York and contributed greatly to the spreading of the Joseph procedure in the USA. Yet another assistant was the American Joseph Safian who reported that up to six doctors from home and abroad were permitted to observe the operations from a platform at the foot of the operating table, for an appropriate payment. They were not permitted to ask questions and there were no explanations and comments on the operative procedure. Was it because Joseph disliked being questioned or because his private patients were being operated upon under local anaesthesia and he did not want any discussion in their presence, giving them the impression that nothing less than 100% of his attention was towards their wellbeing? No one will ever know! To the observers too, whether this exposure was discouraging rather than encouraging and informative, again one will never be able to fathom.

Those who did not know Jacques Joseph personally had an impression that he was a rather bad-tempered and un-cordial teacher. But those who did manage to break through his tough exterior valued his warmth of heart and his humour, and appreciated that there was hidden inside a deeply sensitive man, fascinated by a classical ideal of beauty, who, as a physician, was honestly fond of his unfortunately disfigured patients. Moving testimonials sent to his family in the 1970's and 80's from his disfigured patients and War Veterans long after he died are ample proof of the goodness of his heart and sensitivity of his soul!

Both his surgery and his compassionate view on the subject of aesthetic improvement made him immensely popular and patients gravitated to him from all parts of Europe. This popularity and his consistent good results made him the premier facial plastic surgeon in Europe of his time. Perhaps his greatest contribution is the fact that he systematized corrective, reconstructive and aesthetic rhinoplasty and defined it anew with regard to its aims and techniques. For the distinction of the founder of modern nasal surgery he has perhaps no competition. Both his surgery and his published work have ultimately earned him a place in history as the father of modern facial plastic surgery.[1]

Joseph would have many famous admirers in days to follow, amongst them was Hugo Ganzer, himself an extremely experienced exponent of the Plastic Surgical skills. He was of the view that Joseph was a talented surgeon, who had not only excellent surgical know-how, but also that artistic feeling for form at his disposal. Erich Lexer (1867-1937) too was a fan of his artistic temper and said that everyone who wishes to carry out cosmetic operations must have it.[3] While Joseph had mastered certain chronological operative steps while operating on an injured face or a deformed nose, he would not shy away from newer designs and follow un-trodden roads if the situation demanded. Every defect or disfigurement was thoroughly analyzed before surgery much like the planning session of today. He would plan every step painstakingly never left anything to intuition during the operation. Such was his mastery on the subject of aesthetic and reconstructive Rhinoplasty that even till date some of his methods and some of his results have not been bettered.

### Publications

In 1928 and 1929 the first two sections of Jacques Joseph's book on ‘nasal plastic surgery’ were published at Curt Kabitzsch Publishers, Wuerzburg / Leipzig. This book, Nasenplastik und sonstige Gesichtsplastik nebst Mammaplastik (‘Nasal Plastic Surgery and Other Facial Procedures, and the Plastic Surgery of the Breast’)[4] remains Jacques Joseph's major scientific contribution and was completed in 1931. He had over 30 publications to his credit. Through his monumental work Joseph systematized corrective, reconstructive and aesthetic Rhinoplasty in particular and facial plastic surgery in general and redefined them in their aims and techniques. He emphasized that Rhinoplasty was to address both form and function, and authenticated aesthetic surgery as a serious surgical speciality. His book and his work makes him the unchallenged founder of modern Rhinoplasty and one of the most important pioneers of facial plastic surgery. The book, a masterpiece of 842 pages – which according to Joseph is an ‘atlas and textbook’– is a comprehensive survey of both the principles of analysis and operation planning and techniques for patients with extensive facial wounds. It also deals with the functional and aesthetic indications of Rhinoplasty such as crooked, saddle, and hump nose. Corrective, reconstructive and aesthetic nasal surgery all find a mention as does facial surgery for drooping cheeks, plastic surgery of forehead, jaw, lip, cheek, and lid. The surprise pack is an 80-page appendix on reconstructive breast surgery and mammoplasty techniques. With time and continuing research many of his operative techniques and indications, e.g. resectional septum surgery, have been further developed or changed. Nevertheless, his book ‘*Nasal Plastic Surgery and Other Facial Reconstructive Procedures, With an Appendix on Reconstructive Breast Surgery and Some Other Procedures in the Area of External Plastic Surgery* still remains a landmark of such importance that Rhinoplasty and Facial Plastic Surgery can easily be subdivided into a rather gloomy and ill defined era before Jacques Joseph and a better documented and brighter era after his age and time!

### A doyen of reconstructive plastic surgery

The basic principles of planning skin flaps, suture techniques or wound treatment which Joseph practiced are relevant even today and ought to be understood and followed by every plastic surgeon. He has in his book emphasized the importance of the exact analysis of a deformity or defect and how it is essential to a clear, pre-operative plan. The diagrams and illustrations depicting the general fundamentals of anatomy, facial proportions, the morbid anatomy of a deformity, what is missing and from where, all help the reader to become a better treatment planner. The didactic structure of his book, which illustrates the various causes of each deformity, guides the reader through specific surgical problem situations and their solutions, often in apparently hopeless cases. The language is simple and gives a clear idea of how the author was thinking and encourages the reader to do the same. What we today refer to as looking at the third dimension of a deformity is very clearly apparent. In fact he adds a fourth dimension too by emphasizing the psychological aspect rather boldly. For example, in the chapter “Grounds for rhinomiosis and the relevance of the operation to everyday living”, we read: “One who wishes to be rid of a deformity, who simply wants a ‘normal’ appearance – wishes to be inconspicuous – should not suffer the odium of vanity.”

Not only the author Jacques Joseph but his team of illustrator, photographer and publisher all deserve rich praises for the outstanding publication. The illustrations and pastels are easy to understand, clear and didactically brilliant, making the steps of each procedure and its principles thoroughly comprehensible and enjoyable to read. The balanced and harmonic design, the layout, and the technical excellence visible in the black-and-white photographs all combined can match the best publications of our present day and age. The reprint, using a complex reprographic procedure, is quite comparable in quality to the original and those who possess the book treasure it and pass it on to the next generation with a lot of hope. The original German edition of Joseph's book published in 1931 is a legendary rarity. The English translation by Stanley Milstein was published in 1987. It has 843 pages with 1718 illustrations, some in colour. Bound in Red Morocco leather spine tooled in gold, black cloth boards, and presented in gold-stamped cloth slipcase, it is a collector's dream besides being an outstanding reference book of aesthetic plastic surgery.

Joseph developed many new instruments for his operations, the most well-known being the Joseph's elevator which is still in daily use in operating theatres throughout the world. The book also conveys an understanding of the relationship between operative goal and instrument knowledge. The clever use of novel instruments to suit tricky situations was perhaps his hobby as in his life time he designed many of them.

### Cultural history of the then Germany

This major work of Jacques Joseph is also an impressive reminder of an important, bygone era of medicine. Joseph's career began in Germany at the time of emperor Wilhelm II, brought him the highest recognition as a doctor and ended sadly under the National Socialists with extreme humiliation. In 1933 the National Socialists came to power in Germany and a catastrophe was in the making. Jewish Germans, who were unanimously labelled as ‘dissidents’, were in for a hard time, to put it mildly after so many years. Joseph, being an aristocrat and a jewel amongst Berliners had not taken the increasing Brown danger seriously. Little did he realize that despite the greatest recognition and esteem that he enjoyed in the recent past there would follow - nearly overnight - the deepest official disdain. The Gestapo had engaged the steno typist whom Joseph had employed to finish his textbook, and who lived in his house. He spied on him and blackmailed him on the instructions of his Nazi authorities. The authorities would allow Joseph to carry out only few ‘special approval procedures’. The wave of violent anti-Semitism of the time took a toll on both his practice and his private life.[3]

In Germany today, the importance of Jacques Joseph and his work has largely fallen into oblivion. This is primarily due to the fact that after his death, his achievements, like those of all other Jewish doctors and scientists during the Nazi era, were ignored. After the war, the first publications and praises about Jacques Joseph came from abroad, particularly from his trainees and peers in the USA, where his wife Eleonore and daughter Bella had emigrated. It is thanks to the publisher Dr. Reinhard Kaden and the luxurious reprint of Jacques Joseph's work that this scientific legacy of a great physician is once again accessible to his cynosures.

### Death of an unsung Hero

On 12^th^ February 1934 Jacques Joseph died of a heart attack in the hallway of his house on his way to his practice. Though theories of death by violence or suicide are mostly unfounded, the fact that his myocardial infarction was precipitated by the repeated mishandlings which Joseph suffered at the hands of Nazi thugs can not be ruled out. The German specialist press, already mostly brought into line by the Nazis by then, took no more notice of Joseph's death. Obituaries appeared only in overseas journals and mourners were mostly his trainees and his peers.

### Recognition after death

Jacques Joseph was buried in the Jewish Cemetery in Berlin-Weissensee. After the bombing raid in the Second World War his grave was destroyed and was believed to be no longer identifiable. After much difficult research, it was possible to find the partially buried and overgrown gravestone of Joseph's grave in August 2003. The stone, made of black granite, has since been retrieved, identified and the former inscription deciphered.

**Figure 4:**
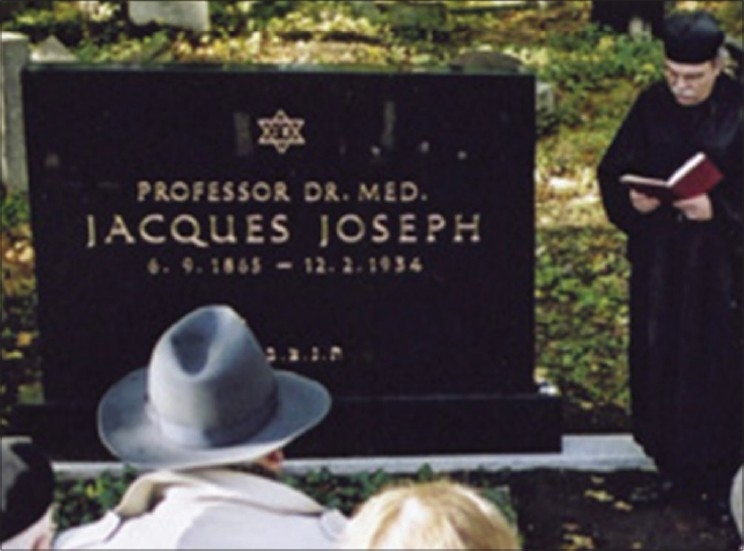
Jacques Joseph's gravestone at the Jewish Cemetery in Berlin-Weissensee. (Source: http://www.jacques-joseph.de/JJ_GB/Grave_GB.html)

Jacques Joseph's gravestone was consecrated on the occasion of the re-erection of his tomb on 17^th^ October 2004, at the same Jewish Cemetery in Berlin-Weissensee. The unveiling and consecration of the stone was conducted by the eminent rabbi Dr. Andreas Nachama in the presence of some 70 guests from home and abroad.[5] Thus, Jacques Joseph was paid the respect which was denied him at his death [Figure 4]. Many private persons and professional associations throughout the world donated generously for the re-erection of his tomb. They included many private citizens and organizations like American Academy of Facial Plastic and Reconstructive Surgery; European Academy of Facial Plastic Surgery; Deutsche Gesellschaft fuer Hals-Nasen-Ohren-Heilkunde, Kopf- und Hals-Chirurgie; Norddeutsche Gesellschaft fuer Otorhinolaryngologie und zervikofaziale Chirurgie; Oto-Laryngologische Gesellschaft zu Berlin; Vereinigung der Westdeutschen Hals-Nasen-Ohren-Aerzte von 1897. The stone, though refurbished, was purposely left unchanged despite the bomb-damage it sustained to reflect the eventful history of the grave. On the front is the new inscription on polished Swedish granite but the back of the stone has been left just as it was when it was found broken, overgrown, damaged, with fragments of inscription. It has been proposed to the Senate that the tomb receives recognition as an official Berlin memorial.[5]

**Figure 2:**
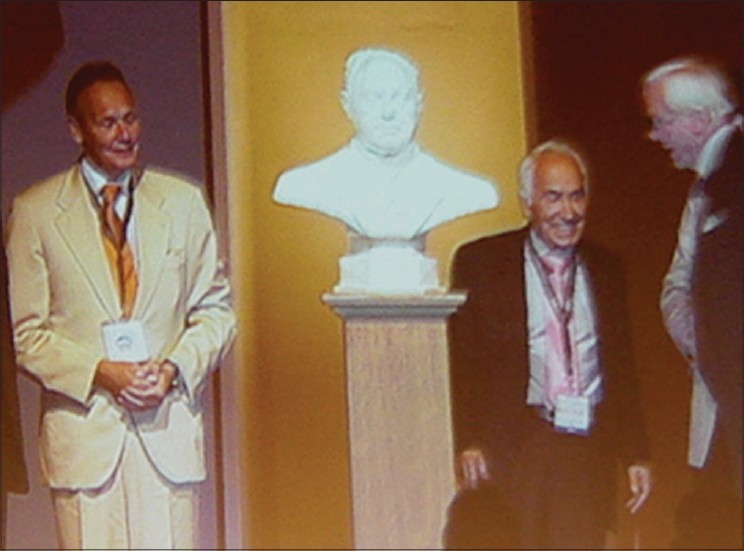
Jacques Joseph being honoured 75 years after his death in the Inaugural Ceremony of the 14th. IPRAS Meeting in Berlin by unveiling of his bust size statue by Prof. Robert Goldwyn, former Editor of PRS. (Source: Author's personal collection)

**Figure 3:**
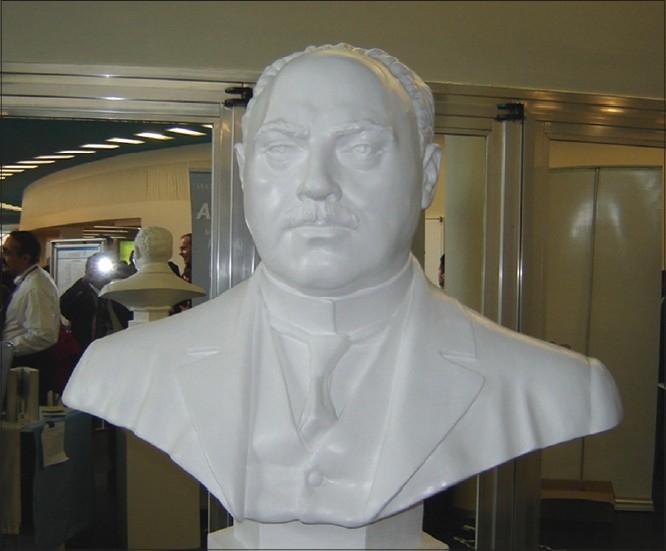
A bust of Jacques Joseph in 14th. IPRAS meeting in Berlin. (Source: Author's personal collection)

When the International Federation of Plastic. Reconstructive and Aesthetic Surgery (IPRAS), met in Berlin for its 14^th^. Congress in June 2007, Jacques Joseph was honoured in its Inaugural Function. Prof. Robert Goldwyn unveiled his bust statue [Figures 2 and 3] and all the dignitaries spoke very highly of him and his contributions to enrich our science, which we all practice and love so passionately.

## References

[1] American Society of Facial Plastic Surgery – History – the father.

[2] Joseph J (1999). Surgical sculptor. Arch Fac Plast Surg.

[3] Behrbohm H, Briedigkeit W, Reintanz G 100 Years of Modern Nasal Surgery – Part 2: The Great Age of Medicine in Berlin.

[4] Joseph J Nasal Plastic Surgeryand Other Facial Reconstructive Procedures, with an Appendix on Reconstructive Breast Surgery and Some Other Proceduresin the Area of External Plastic Surgery. [Nasenplastik und sonstige Gesichtsplastik nebst einem Anhang über Mammaplastik].

[5] Behrbohm H Jacques Joseph's grave reerecter.

